# The Effectiveness of Patient Education Interventions to Oncological Entero-Urostomy Patients and Caregivers: A Small Sample Size Pilot Study

**DOI:** 10.3390/diseases13060164

**Published:** 2025-05-22

**Authors:** Alessandro Spano, Fabrizio Petrone, Emanuele Di Simone, Aurora De Leo, Paolo Basili, Irene Terrenato, Maria Antonietta Picano, Marco Piergentili, Albina Paterniani, Laura Iacorossi, Nicolò Panattoni

**Affiliations:** 1 Department of Biomedicine and Prevention, University of Rome Tor Vergata, 00133 Rome, Italy; alessandro.spano@ifo.it; 2 Nursing Research Unit IFO, IRCCS Regina Elena National Cancer Institute, 00144 Rome, Italy; fabrizio.petrone@ifo.it (F.P.); aurora.deleo@ifo.it (A.D.L.); mariaantonietta.picano@ifo.it (M.A.P.); marco.piergentili@ifo.it (M.P.); nicolo.panattoni@ifo.it (N.P.); 3 Department of Medical, Movement and Wellbeing Sciences, Parthenope University of Naples, 80133 Naples, Italy; emanuele.disimone@uniroma1.it; 4 Clinical Trial Center and Biostatistics and Bioinformatics Unit, IRCCS Regina Elena National Cancer Institute, 00144 Rome, Italy; irene.terrenato@ifo.it; 5 School of Nursing, Sapienza University, IRCCS Regina Elena National Cancer Institute, 00144 Rome, Italy; albina.paterniani@ifo.it; 6 Department of Life, Health and Health Professions Sciences, Link Campus University, 00165 Rome, Italy; l.iacorossi@unilink.it

**Keywords:** patient education, cancer patient, caregiver, entero-urostomy, pilot study

## Abstract

Background: Patient education (PE) is an integral part of treatment from taking charge to the care, assistance, and rehabilitation of the patient, and consists of structured, organised actions, the orientation of which is aimed at finding solutions supported by scientific evidence. Aim: This prospective, descriptive, exploratory, single-centre pilot study aimed to evaluate the effectiveness of a PE intervention for oncological patients with entero-urostomies and their caregivers through the measurement of quality of life, perceived needs, and caregiver burden. Methods: This study was conducted in a National Cancer Institute between 22 December 2022 and 31 March 2023, and it was organised into three specific therapeutic education event days relative to the real needs measured by the patients and caregivers before it. Results: Our results seem to suggest that the PE intervention in entero-urostomy patients improves their quality-of-life levels, while caregivers’ perceived emotional burden levels are reduced. Conclusions: Targeted and individualised PE interventions positively affect self-care and quality of life in patients with an entero-urostomy and the emotional burden perceived by caregivers.

## 1. Introduction

Patient education (PE) is a process of learning and support provided to patients to manage their health condition better [1]. It is a personalised educational approach that provides information, skills, and practical strategies to address the disease, improve quality of life (QoL), and promote patient autonomy. Specifically, for an ostomy patient, it is a fundamental path to help the patient and caregiver understand and adapt to their new condition and image. A structured stoma PE training program using an interactive educational approach to the patient and caregiver can further improve outcomes such as stoma self-management and post-ostomy QoL [2,3,4].

PE can be carried out by various professionals with specific scientific and pedagogical skills, which are learned through dedicated training courses [5]. Furthermore, it involves all phases of the helping relationship, starting from communication of the diagnosis, through a targeted methodology, up to the ability to dynamically cope with the difficulties that the disease brings with it [6]. Specifically, the nurse plays a preponderant role in the patient’s educational process, so much so that PE is considered a fundamental component of nursing [7,8].

Ostomy cancer patients and their caregivers have special needs that require personalised and continuous care [6]. Stoma patients and their caregivers experience physical, psychological, and emotional stress, and an economic burden. A new body image, difficulties in social relationships, anxiety, depression, embarrassment, and sexual problems can affect this specific population [3,7]. For these reasons, PE interventions must be implemented in oncology centers, which can overcome the misinformation of patients and caregivers and all the barriers that may in some way hinder their ability to receive, process, and put the information into practice. When implementing a PE process, it is essential that the health information provided, in addition to being of high quality, is easily interpretable by patients. In the literature, we can find various tools to support the educational methodology, always and in any case built with an easily understandable language, for example, by integrating diagrams, images, or multimedia supports [9,10,11].

The educational approach in PE can be practical or theoretical and can make use of multiple expository (academic lessons), operational (learning by doing), and investigative (problem-based learning) methodologies.

The Stoma care Nurse Specialist (ScNS) is the point of reference for ostomy patients, providing them with specialised support and care. These professionals play a key role in implementing interventions aimed at the prevention, treatment, rehabilitation, and education of ostomy patients. Working closely with patients, the ScNS provides detailed information on daily management of the ostomy, including care of the stomal area, application of collection bags, and adoption of a proper lifestyle. It also enables the ScNS to assess the patient’s specific needs, provide individualised support, and prepare an appropriate care plan in preparation for surgery and the postoperative period [6]. The presence of an ScNS in the care pathway contributes significantly to improving QoL and promoting appropriate treatment adherence by ensuring ongoing support and effective ostomy management [12]. Our PE project originated as a nursing reflection on personalised patient care. PE is also a quality requirement from the OECI (Organization European Cancer Institute) accreditation system for Comprehensive Cancer Centers, such as the Istituto Ricovero e Cura a Carattere Scientifico (IRCCS) National Cancer Institute “Regina Elena” in Rome, Italy.

Thus, PE is an integral part of treatment, from taking charge to the care, assistance, and rehabilitation of the patient, and consists of structured, organised actions, the orientation of which is aimed at finding solutions supported by scientific evidence [6,13]. Promoting learning processes is a stimulating objective as it puts the operator in a strong relationship with the patient and caregiver. The “educational” approach aims to provide knowledge and ensure understanding of health problems in such a way as to encourage autonomous analysis of the patient’s behaviours and habits and to make informed decisions for their health [13]. Indeed, further research seems necessary to underline the effectiveness of patient education interventions for oncological entero-urostomy patients and caregivers.

To this end, we hypothesised that performing PE on patients with an entero-urostomy will improve their levels of QoL, while their levels of perceived emotional burden will be reduced for caregivers.

Therefore, this study aimed to evaluate the effectiveness of the PE intervention for oncological patients with entero-urostomies and their caregivers through the measurement of QoL, perceived needs, and caregiver burden.

## 2. Materials and Methods

### 2.1. Study Design

A prospective, descriptive, exploratory, single-centre pilot study [14] was conducted at the IRCCS National Cancer Institute “Regina Elena” in Rome, Italy between 22 December 2022 and 31 March 2023. The study was conducted following Strengthening the Reporting of Observational Studies in Epidemiology (STROBE) guidelines [15] (Table S1).

### 2.2. Patient Education Intervention Methodology

According to Patient Education Guidelines [16], the PE intervention was organised into three specific therapeutic education events days, in which the educational contents were calibrated based on the real needs measured by the patients and caregivers assessed before it with the Needs Evaluation Questionnaire (NEQ) (detailed below).

Each PE day was conducted by a staff of three ScNSs, previously trained in PE, with a specific training course [5].

The learning areas were divided into three for each PE event day [16]: PE event day one based on ostomy theoretical knowledge (a cognitive learning area relating to knowledge, concepts, procedures, and principles); PE event day two based on gesturality knowledge (a gestural learning area relating to operational and manual skills); and the third and last PE event day based on socio-relational knowledge (a relational learning area related to social, family, and work relationships).

On all days, there was a mix of face-to-face knowledge transmission with interactive reinforcement questions (a participatory lesson where an expert “explains” and the “learners” listen and interact through an exchange of information/opinions) during which patients and caregivers could comment extemporaneously but anonymously through a digital platform; the questions were read aloud to the group, forming the basis of discussion on which to build new knowledge to be transferred.

Finally, each PE event day lasted approximately 2 h, and the patient and caregivers’ recruitment were free of a fee and voluntary.

At the end of the last PE event day, Conversation Maps, specially constructed during the events, were left for patients as a reference guide for home self-management.

In particular, Conversation Maps are an educational and didactic tool. They consist of quick consultation image guides through which people are engaged in discussion, and share doubts and experiences about their lives to reinforce the theoretical concept acquired [17].

### 2.3. Patient Recruitment

To achieve our aim, a consecutive sample of 10 participants (five patients and five caregivers) was enrolled. All these participants had an oncological entero-urostomy clinical pathway at the IRCCS National Cancer Institute “Regina Elena” in Rome, Italy. Specifically, we considered all patients who accessed the Stoma Care Nurse Specialist’s Outpatient clinic. Enrollment in the study was based on specific inclusion criteria as follows: (i) aged > 18 years; (ii) patient having a histologically proven diagnosis of cancer; (iii) patient having entero-urostomies; (iv) formal or informal caregiver of a patient having entero-urostomies; (v) availability to provide written informed consent to study participation; and (vi) willingness to participate in several PE meetings both for patients and caregivers.

The patients without a histologically proven cancer diagnosis or with cognitive deficits or pathological conditions that may be an obstacle to active participation in the study were excluded.

### 2.4. Data Collection and Assessment Tools

All patients recruited into the study were invited to complete questionnaires at three different time points: before the PE event (t0), at the end of the training event (t1), and 30 days after the event itself (t2). We collected the following data: (1) sociodemographic (age, gender, education, marital status, work activity, and family members) and clinical data (pathology, date of diagnosis, previous therapy, and ongoing therapy); (2) Needs Evaluation Questionnaire (NEQ) [18]: a validated tool created for analysis of the needs of cancer patients, which has proven useful in obtaining a systematic and undistorted vision of patients’ needs; (3) Short-Form Health Survey 36, SF-36 [19]: this instrument aims to explore your concept of well-being related to the dimensions of physical, psychological, and emotional well-being to assess QoL overall; and (4) Ostomy Self-Care Index (OSCI) [20]: a validated tool that measures self-care in people with an ostomy and is composed of 32 items and four sections (A, B, C, and D) that respectively evaluate self-care maintenance, self-care monitoring, self-care management, and self-care confidence. The score has a theoretical range between 31 (worst possible self-care) and 165 (optimal self-care).

Moreover, all caregivers recruited in the study were invited to complete specific questionnaires at the same time points t0, t1, and t2. We collected the following information: (1) sociodemographic data (age, gender, education, marital status, work activity, and family members); (2) Caregiver Need Assessment (CNA) [21]: a tool with good internal consistency that measures the needs perceived by the caregiver relating to the care of their family member. It is made up of 17 items (total score: 0–51), which refer to needs that can be grouped into two factors: need for emotional and social support (α = 0.765), and information and communication needs (α = 0.742). For each item, there is a Likert scale with a score between zero and three (not at all, a little, quite a lot, and a lot): a higher score corresponds to a greater intensity of the perceived need; (3) Zarit Burden Interview [22]: it is a validated scale aimed at ascertaining the subjective tension overload (burden) to which a caregiver is subjected. The original version of the scale and the one validated in Italian are made up of 22 items. Conceived in the field of diseases of the nervous system and psychiatry, it has also been applied in other fields, such as oncology.

Finally, all patients and caregivers will be given a satisfaction questionnaire regarding the PE event at the end of it based on a five-point Likert scale.

### 2.5. Statistical Analysis

Following the study design [14], descriptive statistics were used to summarise relevant information about the study and presented as mean ± standard deviation (SD) or frequencies and percentage values. Statistical analyses were performed with SPSS 29.0 software (SPSS, Chicago, IL, USA).

### 2.6. Ethical Considerations

This study was conducted according to the Declaration of Helsinki [23] and approved by the Health Direction of IRCCS National Cancer Institute “Regina Elena” and the Local Ethics Committee IRCCS Lazio (No. 1808/22 of 13 December 2022). The data collected were analysed in aggregate and anonymously and confidentiality coded to guarantee participants’ privacy. The recruitment was free and voluntary, and after written informed consent was signed, and at any time, the participants enrolled could leave the study without consequences in the clinical pathway. All the nurses involved in the study completed a PE training course.

## 3. Results

A total of 10 participants (five oncological entero-urostomy patients and five caregivers) were included in the study. The sociodemographic and clinical characteristics are summarised in Table 1. The sample was mainly composed of predominantly male patients and predominantly female caregivers, both 60%, and all married to each other (100%). Furthermore, patients from 60% to 80% were undergoing chemotherapy or radiotherapy treatment.

Regarding patient surveys, the NEQ’s results showed an overall increase in patients’ perceived needs at t2 compared with the baseline survey.

Similarly, Table 2 describes the results for the OSCI showing the average increase in several dimensions of self-care. The average trend of the OSCI score shows how for all the self-care dimensions explored (self-care maintenance, self-care monitoring, self-care management, and self-care confidence), we have a decline at t1 and then an increase at t2 tending to optimal self-care.

**Figure 1:**
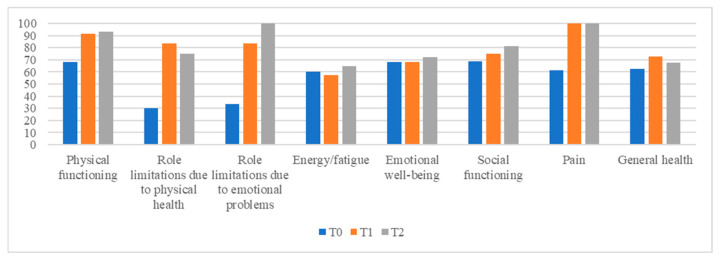
SF-36 results.

Figure 1 graphically shows the trend in QoL related to the dimensions of physical, psychological, and emotional well-being from t0 to t2. All the dimensions explored except for “role limitations due to physical health”, “pain”, and “general health” show a trend of improvement at t2.

From a caregiver’s point of view, the CNA results show an overall increase from t0 to t2, reporting an increase in the needs perceived by the caregiver relating to the care of their family member (Table 3), while the overall Zarit Burden Interview results are decreasing across follow-ups, reflecting a decrease in the subjective tension overload (burden) to which the caregiver is subjected (Table 4).

The levels of satisfaction for each PE event are encouraging as they are high, very close to the maximum value of 5 (4.81 on average for patients and 4.71 for caregivers).

## 4. Discussion

The results achieved by this prospective, descriptive, exploratory, single-centre pilot study made it possible to achieve the study aim, which was aimed at evaluating the effectiveness of the PE intervention for patients with entero-urostomies and their caregivers through the measurement of QoL and perceived needs before the events (t0), at the end of the PE intervention (t1), and 30 days after the PE intervention (t2).

Further, we can state that these results seem to suggest that performing a PE intervention on patients with an entero-urostomy could improve their levels of QoL, while their levels of perceived emotional burden will be reduced for caregivers.

Specifically, regarding the actual needs detected with the patient NEQ, the results show an apparent increase in need from t0 to t1, which returns to generally adequate levels at t2. For example, needs such as “more information” or “involvement in treatment choices,” “more attention from health care staff”, or “need for reassurance”, “feeling more useful in the family”, or “being less left to one’s own devices” show an increasing trend at t1 that tends to improve after the last day of PE.

In parallel, the caregiver’s perceived needs related to their family member’s care overlap. In particular, needs such as “need to be informed”, “be empowered to deal with the care needs of the family member”, “be prepared to deal with changes in the family”, and “need to compare myself with family members of other patients” show an increasing trend across PE days.

This trend can be accounted for by an increase in theoretical awareness of an ostomy and its practical management, which initially increased difficulties in the middle phase of the study, growing doubts in both patients and caregivers.

Scores related to self-care, in all its explored dimensions, also show the same trend. Moreover, strongly supporting the initial hypothesis are the results related to caregiver burden, which show substantial decreases in all items except the one inherent in the question, “Do you fear what the future holds for your family member?”, and the results related to QoL show substantial improvements in all dimensions explored. We believe that this decline can be justified by the oncological disease and not by the stoma.

Our results are in line with other similar educational events reported in the literature, which show high levels of patient satisfaction in participating in such events with an important contribution to improving self-care and QoL levels [24,25,26]. Carrying out PE training interventions enables the patient to manage their treatment process (empowering patients) as many people wish to play an active role in protecting their health: when they are fit, they want to know how to protect and improve their condition; and when they are sick, they ask about treatment options and the chances of success. So, in addition to seeking quick and effective advice when they need it, people want to know what they can do to help themselves [27]. Furthermore, the training carried out by the ScNS may also have positively influenced the participants’ satisfaction with the training event. Studies underline how essential it is to be trained by specialised reference figures for both patients and caregivers [28] throughout the treatment process. This presence influences the perception of empathy, honesty, and emotional support [29], and on the best possibility of communicating and sharing information in the decision-making process [30]. The use of Conversation Maps may also have contributed to organising, synthesising, and deepening new knowledge, and, therefore, may have influenced the levels of satisfaction of patients and caregivers; studies report, in fact, their important support as a PE tool [31].

However, the literature agrees on the fact that the feasibility of patient PE programs requires the creation of dedicated tools and an effort to both empower patients and support healthcare professionals [24,32], with positive long-term impacts on healthcare costs [33].

### Limitations

The authors are aware of the finding’s limitations. The limited sample size and the pilot design of the study did not allow us to carry out inferential statistical analyses and to generalise the results. The research aims to test the effectiveness of a targeted PE pathway on a small oncological population based on the specific needs detected [14]. Thus, this study aimed to test PE methodology and our effectiveness hypothesis before starting with more complex research (biggest sample, longest timing observation, other tools, etc.). Further studies will be needed to conduct the PE model on a larger population and in multicentric studies to obtain generalizable results. The improvement trend in outcomes for both patients and caregivers suggests the feasibility of such PE events. A future qualitative study dedicated to exploring the experiences of patients, carers, and healthcare professionals will address the shortcomings of the present descriptive study.

## 5. Conclusions

Despite the limitations mentioned above, the objective of the study was achieved. Targeted and individualised interventions on PE have positive effects on self-care and QoL in cancer patients with an entero-urostomy and on the emotional burden perceived by caregivers.

Our results could suggest the usefulness of specific PE training for healthcare professionals and encourage the construction of methodologically appropriate and individualised PE events on patients with a stoma, who expressed high levels of satisfaction in our attempt.

To conclude, future research in this field should be conducted on a larger population and from a multicentre perspective in contexts other than oncology, considering our results as a valid reference basis.

## Figures and Tables

**Table 1:** Sample sociodemographic and clinical characteristics.

	Patients N (%)	Caregivers N (%)
**Total**	5 (100%)	5 (100%)
**Gender**		
Male	3 (60%)	2 (40%)
Female	1 (20%)	3 (60%)
Unknown	1 (20%)	0
**Education**		
Primary	1 (20%)	0
Secondary	1 (20%)	3 (60%)
Higher	2 (40%)	2 (40%)
Degree	1 (20%)	0
**Marital status**		
Married	5 (100%)	5 (100%)
Unmarried	0	0
**Job**		
Worker	1 (20%)	1 (20%)
Housewife	1 (20%)	2 (40%)
Retired	2 (40%)	2 (40%)
Undeclared	1 (20%)	0
**Living alone**		
Yes	0	0
No	5 (100%)	5 (100%)
**Children**		
Yes	4 (80%)	5 (100%)
No	1 (20%)	0
**Surgery**		
Yes	5 (100%)	/
No	0	/
**Chemotherapy**		
Yes	4 (80%)	/
No	1 (20%)	/
**Radiotherapy**		
Yes	3 (60%)	/
No	2 (40%)	/

**Table 2:** OSCI results.

	T0	T1	T2
Pz 1	134	135	147
Pz 2	153	123	145
Pz 3	143	136	152
Pz 4	142	126	144
Pz 5	151	128	148
	***Mean (SD)***	***Mean (SD)***	***Mean (SD)***
Overall	144.6 (±0.4)	129.6(±0.1)	147.7 (±0.1)

**Table 3:** CNA results.

Question n°	T0	T1	T2
	Mean (SD)	Mean (SD)	Mean (SD)
1	3.20 (±1.30)	2.60 (±1.14)	3.60 (±0.55)
2	2.80 (±1.64)	2.80 (±1.10)	3.20 (±0.45)
3	2.80 (±1.64)	2.20 (±1.30)	3.60 (±0.55)
4	1.80 (±1.30)	2.00 (±1.22)	1.80 (±1.30)
5	2.20 (±1.10)	2.40 (±0.55)	3.20 (±0.45)
6	2.20 (±1.64)	1.60 (±0.55)	1.40 (±0.55)
7	2.00 (±1.00)	3.40 (±0.55)	3.80 (±0.45)
8	2.20 (±1.64)	2.60 (±0.55)	3.40 (±0.55)
9	2.40 (±1.34)	1.60 (±0.89)	1.60 (±0.89)
10	2.80 (±1.30)	2.60 (±1.14)	3.40 (±0.55)
11	2.00 (±1.00)	1.60 (±0.55)	1.40 (±0.55)
12	2.20 (±1.10)	3.40 (±0.89)	3.80 (±0.45)
13	1.80 (±0.84)	2.60 (±1.14)	3.40 (±0.55)
14	1.00 (±0)	1.60 (±1.34)	1.80 (±1.30)
15	1.40 (±0.55)	2.80 (±1.30)	3.00 (±1.00)
16	1.00 (±0)	1.00 (±0)	2.20 (±0.84)
17	1.00 (±0)	1.40 (±0.55)	1.40 (±0.55)

Note: Range 1–4 (1. Never, 2. Sometimes, 3. Often, 4. Always).

**Table 4:** Zarit Burden Interview results.

Question N°	T0	T1	T2
	*Mean (SD)*	*Mean (SD)*	*Mean (SD)*
1	0.20 (±0.45)	0.60 (±0.55)	0 (±0)
2	1.00 (±0.71)	0.60 (±0.55)	1.20 (±0.84)
3	1.60 (±0.89)	0 (±0)	0 (±0)
4	0.20 (±0.45)	0 (±0)	0 (±0)
5	0 (±0)	0 (±0)	0 (±0)
6	0.80 (±0.84)	0 (±0)	0 (±0)
7	1.80 (±1.10)	1.40 (±0.55)	2.20 (±0.84)
8	0.60 (±0.89)	0 (±0)	0.60 (±0.89)
9	1.20 (±1.10)	0.40 (±0.55)	0.40 (±0.55)
10	0.80 (±0.84)	0.40 (±0.55)	0.80 (±0.84)
11	0 (±0)	0 (±0)	0 (±0)
12	1.40 (±1.67)	0.40 (±0.55)	0.40 (±0.55)
13	0.60 (±0.55)	0 (±0)	0 (±0)
14	0.80 (±1.30)	0 (±0)	0.40 (±0.55)
15	0.40 (±0.89)	0.60 (±0.55)	0.60 (±0.55)
16	0 (±0)	0.40 (±0.55)	0.20 (±0.45)
17	0.40 (±0.55)	0.60 (±0.55)	0.40 (±0.55)
18	0 (±0)	0 (±0)	0 (±0)
19	0.40 (±0.55)	0.60 (±0.55)	0 (±0)
20	1.40 (±1.14)	1.00 (±1.00)	0.40 (±0.55)
21	1.00 (±1.41)	0 (±0)	0 (±0)
22	1.40 (±0.55)	0 (±0)	0 (±0)

Note: Range 0–4 (0. Never, 1. Rarely, 2. Sometimes, 3. Often, 4. Always).

## Data Availability

The datasets generated and/or analysed during the current study are not publicly available but are available from the corresponding author on reasonable request due to restrictions privacy and ethical issues.

## Supplementary Materials

The following supporting information can be downloaded at https://www.mdpi.com/article/10.3390/diseases13060164/s1, Table S1: STROBE Statement—Checklist of items that should be included in reports of cohort studies.
